# Legionellosis Outbreaks Associated with Two Hotels — U.S. Virgin Islands, October 2024–April 2025

**DOI:** 10.15585/mmwr.mm7525a2

**Published:** 2026-07-02

**Authors:** Sarah Gallalee, Hannah M. Cranford, Aubrey Drummond, Lisa LaPlace Ekpo, Brett R. Ellis, Esther M. Ellis

**Affiliations:** ^1^Epidemic Intelligence Service, CDC; ^2^U.S. Virgin Islands Department of Health.

**Summary**

**What is already known about this topic?**

Legionellosis is a bacterial disease caused by inhalation or aspiration of *Legionella* bacteria. *Legionella* bacteria can pose a health risk when they contaminate building water systems.

**What is added by this report?**

In November 2024, two outbreaks of legionellosis occurred at two hotels in the U.S. Virgin Islands. Two of four total patients with legionellosis were hospitalized; none died. Although legionellosis outbreaks are commonly associated with warm water sources, probable sources of exposure included both cold (unheated) and hot (heated) water from showerheads and sinks in guest rooms.

**What are the implications for public health practice?**

Implementing effective water management programs and ensuring adequate water system disinfection to prevent the growth of *Legionella* bacteria is important for hotel operators.

## Abstract

Legionellosis is a bacterial disease caused by inhalation or aspiration of *Legionella *bacteria; Legionnaires disease is a type of legionellosis characterized by illness with pneumonia. During November 2024, the U.S. Virgin Islands (USVI) Department of Health (VIDOH) was notified of two confirmed Legionnaires disease cases among travelers to two different hotels on St. Croix Island. VIDOH investigated to determine exposure sources and prevent additional cases. Two additional legionellosis cases were identified. The four patients with cases were aged 53–73 years; two patients were hospitalized and none died. At hotel A, *L. pneumophila* was detected in three of 21 (14%) environmental samples. VIDOH required hotel A to close one guest room, remediate, and retest. At hotel B, *L. pneumophila* was detected in 22 of 40 (55%) samples. VIDOH required hotel B to cease hotel operations until remediation and retesting were completed. *L. pneumophila* was isolated from shower samples at both hotels, in the cistern and cold water system at hotel A, and in cold and hot water systems at hotel B. The two USVI outbreaks underscore the importance of reporting legionellosis among returned travelers to facilitate local public health investigations and prevent additional cases. In addition, in tropical climates, cold water systems operate at temperatures favorable for *Legionella* growth (77°F–113°F [25°C–45°C]), highlighting the importance of effective water management programs and water system disinfection to prevent disease spread.

## Investigation and Results

### Hotel A

On November 6, 2024, CDC notified VIDOH of a case of Legionnaires disease confirmed by a urinary antigen test (UAT) in a man aged 73 years (patient 1); Council of State and Territorial Epidemiologists case definitions for legionellosis were used. On October 30, the patient developed fever, shortness of breath, body aches, joint pain, neck pain, loss of appetite, and diarrhea. He was hospitalized in his U.S. state of residence after returning from travel to St. Croix Island and recovered. He reported staying in two guest rooms at hotel A during October 21–29.

During the investigation of patient 1, a case of probable legionellosis was identified in a woman aged 71 years (patient 2). Patient 2 was a travel companion of patient 1, shared the same travel history, stayed in the same guest rooms, and experienced symptoms including fever and body aches, beginning November 4. She received treatment with antibiotics but was not tested for legionellosis. Both patients reported using showers and sinks in the guest rooms and reported no other water exposures.

In response to these cases, VIDOH conducted on-site environmental sampling at hotel A.* Sampling consisted of 21 swab and bulk water samples from the two guest rooms (showers and sinks), the water heater supplying those guest rooms, a cistern (container that collects and stores rainwater), and the water supply pipe separate from the cistern (Table). In the guest rooms, the bulk water samples were drawn immediately when the faucet or showerhead was turned on to capture a cold (unheated) water system sample and after running the water until hot to capture a hot (heated) water system sample. The cistern and water supply pipe were part of the cold water system. Samples were submitted to an external laboratory for *Legionella* species testing using Legiolert.^†^ *L. pneumophila* was detected in one showerhead, the cistern, and the water supply pipe. Serogroup 1 was detected in one sample and serogroups 2–14 in two samples. The most probable number (MPN) per 10 mL ranged from 1.1 to 213.3, indicating uncontrolled *Legionella* bacterial growth^§^ (*1*). All three positive samples were collected from cold (unheated) water system sources with water temperatures of 80°F–87°F (26.7°C–30.6°C). Free chlorine levels ranged from undetectable (<0.02 mg/L) to 0.04 mg/L in guest rooms, 0.03 in the cistern, and 0.02 in the water heater.

**TABLE:** *Legionella pneumophila* environmental sampling and culture results from an investigation of two unrelated legionellosis outbreaks at two hotels — U.S. Virgin Islands, November 2024

Sampling site	Sample type*	Water temperature, °F (°C)	Free chlorine, mg/L	*Legionella pneumophila* culture result	Serogroup	MPN per 10 mL
**Hotel A**						
Guest room 1						
Kitchen sink	Swab	—	—	NG	—	—
	Bulk, cold	71 (21.7)	0	NG	—	—
	Bulk, hot	142 (61.1)	0	NG	—	—
Bathroom sink	Swab	—	—	NG	—	—
	Bulk, cold	75 (23.9)	0.04	NG	—	—
	Bulk, hot	149 (65.0)	0.04	NG	—	—
Bathroom shower	Swab	—	—	NG	—	—
	Bulk, cold	80 (26.7)	0	*L. pneumophila*	2–14	14.6
	Bulk, hot	150 (65.6)	0	NG	—	—
Guest room 2						
Bathroom sink	Swab	—	—	NG	—	—
	Bulk, cold	79 (26.1)	0	NG	—	—
	Bulk, hot	149 (65.0)	0	NG	—	—
Bathroom shower	Swab	—	—	NG	—	—
	Bulk, cold	79 (26.1)	0	NG	—	—
	Bulk, hot	148 (64.4)	0	NG	—	—
Hot water heater	Swab	—	—	NG	—	—
	Bulk, hot	—	0.02	NG	—	—
Cistern	Swab	—	—	NG	—	—
	Bulk, cold	85 (29.4)	0.03	*L. pneumophila*	2–14	1.1
Pipe	Swab	—	—	NG	—	—
	Bulk, cold	87 (30.6)	0.14	*L. pneumophila*	1	213.3
**Hotel B**						
Guest room 1						
Kitchen sink	Swab	—	—	*L. pneumophila*	1	3.9
	Bulk, cold	73 (22.8)	0.45	NG	—	—
	Bulk, hot	105 (40.6)	0.45	*L. pneumophila*	1	22.3
Bathroom sink	Swab	—	—	NG	—	—
	Bulk, cold	70 (21.1)	0.25	NG	—	—
	Bulk, hot	103 (39.4)	0.25	*L. pneumophila*	1	47.4
Shower	Swab	—	—	*L. pneumophila*	1	149.0
	Bulk, cold	90 (32.2)	0.28	NG	—	—
	Bulk, hot	110 (43.3)	0.28	NG	—	—
Guest room 2						
Kitchen sink	Swab	—	—	*L. pneumophila*	1	1.1
	Bulk, cold	86 (30.0)	0.28	*L. pneumophila*	1	26.4
	Bulk, hot	114 (45.6)	0.28	*L. pneumophila*	1	3.9
Bathroom 1 sink	Swab	—	—	NG	—	—
	Bulk, cold	84 (28.9)	0.18	NG	—	—
	Bulk, hot	109 (42.8)	0.18	*L. pneumophila*	1	72.3
Bathroom 1 showerhead	Swab	—	—	*L. pneumophila*	1	18.7
	Bulk, cold	88 (31.1)	0.25	NG	—	—
	Bulk, hot	105 (40.6)	0.25	*L. pneumophila*	1	21.9
Bathroom 1 bath faucet	Swab	—	—	NG	—	—
	Bulk, cold	88 (31.1)	0.87	*L. pneumophila*	1	41.6
	Bulk, hot	107 (41.7)	0.87	*L. pneumophila*	1	72.3
Bathroom 2 sink	Swab	—	—	*L. pneumophila*	1	1.1
	Bulk, cold	83 (28.3)	0.79	NG	—	—
	Bulk, hot	112 (44.4)	0.79	*L. pneumophila*	1	78.8
Bathroom 2 showerhead	Swab	—	—	*L. pneumophila*	1	126.9
	Bulk, cold	83 (28.3)	0.04	*L. pneumophila*	2–14	1.1
	Bulk, hot	102 (38.9)	0.04	*L. pneumophila*	2–14	2.2
Guest room 3						
Kitchen sink	Swab	—	—	*L. pneumophila*	1	105.7
	Bulk, cold	82 (27.8)	0.47	*L. pneumophila*	1	92.1
	Bulk, hot	110 (43.3)	0.47	*L. pneumophila*	2–14	47.4
Bathroom sink	Swab	—	—	NG	—	—
	Bulk, cold	80 (26.7)	0.05	NG	—	—
	Bulk, hot	82 (27.8)	0.05	NG	—	—
Shower	Swab	—	—	*L. pneumophila*	1	22.3
	Bulk, cold	83 (28.3)	0.08	NG	—	—
	Bulk, hot	118 (47.8)	0.08	*L. pneumophila*	1	65.9
Air conditioning unit	Swab	—	—	NG	—	—
Cistern	Bulk, cold	—	0	NG	—	—
Hot water heater	Bulk, hot	130 (54.4)	—	NG	—	—
	Bulk, hot	130 (54.4)	—	NG	—	—

**Abbreviations:** MPN = most probable number; NG = no growth.  * Bulk samples were either drawn immediately when the faucet or showerhead was turned on (bulk, cold) to capture the cold (unheated) water system or after running the water until hot (bulk, hot) to capture the hot (heated) water system.

### Hotel B

On November 22, 2024, a member of the public notified VIDOH of a woman aged 53 years (patient 3) who was hospitalized in an intensive care unit in her U.S. state of residence after returning from travel to St. Croix Island, where she had stayed at hotel B. Patient 3 had Legionnaires disease with laboratory confirmation by UAT. She had stayed in three guest rooms at hotel B during October 31–November 9. On November 7, she experienced chest tightness and trouble breathing. After returning to her state of residence, she was hospitalized and received a diagnosis of severe sepsis, bilateral pneumonia, and acute respiratory failure. During the investigation of patient 3, a probable case of Legionnaires disease was identified in another woman aged 55 years (patient 4), who was a family member of patient 3 and had traveled with her, had stayed in the same guest rooms, had symptoms consistent with Legionnaires disease (shortness of breath, cough, fever, headache, and muscle aches), and had received a positive serologic test result detecting antibodies to *L. pneumophila*. She had been treated with antibiotics for her illness, and completely recovered. Three additional family members who traveled with patient 3 also felt ill and received testing by UAT; all results were negative.

VIDOH collected 40 swab and water samples from showers, sinks, and faucets in the three guest rooms, cistern, and water heater (Table) at hotel B.^¶^ In the guest rooms, the bulk water samples were drawn immediately when each water source was turned on to obtain a sample from the cold water system and after running the water until hot to obtain a sample of the hot water system. The cistern was part of the cold water system only. Samples were sent for *Legionella* species testing at the same laboratory that had tested hotel A samples. *L. pneumophila* was detected in 22 (55%) samples, including samples from six sinks and four showers. The positive samples consisted of 19 from serogroup 1 (86%) and three from serogroups 2–14 (14%). MPNs per 10 mL ranged from 1.1 to 149, indicating uncontrolled *Legionella* bacterial growth. Positive water samples were taken from a mixture of cold (unheated) and hot (heated) water sources, with temperatures ranging from 82°F to 118°F (27.8°C to 47.8°C). Free chlorine levels ranged from 0.04 to 0.87 mg/L among samples from cold and hot water sources from sinks and showers. The free chlorine level was below the detectable limit in the cistern and was not measured in the water heater because of limited accessibility. This activity was reviewed by CDC, deemed not research, and conducted consistent with applicable federal law and CDC policy.**

## Public Health Response

VIDOH launched an investigation to determine possible sources of infection, mitigate exposures, and educate hotel staff members and guests, health care providers, and the public. Both hotels temporarily closed implicated water systems and undertook remediation and response activities to control the growth of *Legionella*. The hotels worked with VIDOH to identify and contact guests who had stayed in the identified guest rooms at the properties within approximately 4 weeks before identification of each outbreak. Guests were notified of potential *Legionella* bacteria exposure and advised to monitor themselves for cough, fever, and shortness of breath; to seek medical attention if symptoms developed; and to inform their health care provider about the exposure to aid in timely testing, diagnosis, and treatment.

VIDOH established a dedicated outbreak telephone hotline to address public concerns, provide information, and offer guidance to persons who might have been exposed. An official VIDOH press release was issued to announce the hotel B outbreak and inform the public of the ongoing investigation and public health actions (*2*). The press release emphasized the importance of recognizing symptoms early and encouraged potentially exposed persons to consult their health care providers.

VIDOH required hotel A to close the guest room where patients 1 and 2 had stayed. VIDOH provided recommendations to remediate the hotel’s water system and conducted follow-up testing to confirm the absence of *Legionella* bacteria in the system, in accordance with CDC guidance (*1*). The property owner conducted remediation during November 2024–February 2025, including replacing the showerhead and plumbing, hyperchlorinating the system, evaluating filtration, permanently closing the cistern with *Legionella* bacteria growth, and creating an access point for adding disinfectant to water piping. Postremediation sampling was conducted during January–February; hotel A was then cleared to fully reopen after test results indicated that the water system was well controlled.

VIDOH required hotel B to close the entire hotel until remediation and retesting for *Legionella* bacteria were completed. VIDOH guided the hotel in remediating the plumbing system and guest rooms. The property owner completed remediation during November 2024–April 2025, including replacing plumbing and fixtures, hyperchlorinating the system, and evaluating filtration. Sampling was conducted in January and April; after testing no longer detected *Legionella *bacteria, hotel B was cleared to fully reopen.

## Discussion

VIDOH’s investigation of two unrelated legionellosis outbreaks at two hotels highlighted *Legionella* environmental challenges, transmission patterns, and case detection limitations in tropical climates. At hotel B, both cold and hot water systems were implicated; at hotel A, only the cold water system had detectable bacteria. Although hot water systems typically have temperatures that favor *Legionella* growth (77°F–113°F [25°C–45°C]), elevated cold water system temperatures can also increase the risk for colonization (*3*,*4*). In tropical climates such as those in USVI, consistently warmer temperatures can create ideal conditions for bacterial proliferation in cold water systems. These findings highlight the need for tailored *Legionella* bacteria control guidance for warmer environments.

At hotel B, multiple water samples tested positive for *Legionella* bacteria at temperatures above the optimal range for growth (>113°F [>45°C]), indicating the bacteria’s persistence under a wide range of temperatures. Hotel B’s hot water system might not have reached temperatures sufficiently high to suppress growth of *Legionella* bacteria. Water management programs should include protocols to maintain hot water storage >140°F (>60°C) and circulation >120°F (>49°C) to reduce the risk for *Legionella* growth (*1*). These findings highlight the observation that within a water system lacking thermal control, the system relies entirely on disinfectant to control *Legionella* bacterial growth. Both hotels had water samples with free chlorine levels that were below the detectable limit (eight samples at hotel A and one at hotel B).

Both hotels used mixed water supply systems, with combinations of cisterns, municipal water, and private bulk sources (e.g., water trucks). Cisterns in USVI are large volume storage containers typically built into the foundations of buildings to collect and store untreated rainwater captured on the roof. If not properly maintained, cisterns can harbor pathogens (*5*). Cisterns pose challenges for cleaning, monitoring, and disinfectant dosing and risk recontamination from open connections. Ongoing improvements in maintenance and disinfection recommendations for these systems are needed (*1*).

Legionnaires disease has a low attack rate (1%–6%) (*6*), and potable water outbreaks typically involve persons who were exposed to the same facility at different times. In these two outbreaks, additional legionellosis cases were detected among family members who traveled together, demonstrating clustering associated with shared exposures in guest rooms or specific showers. Lower respiratory specimens were not available for any patients; therefore, molecular comparisons with environmental results were not possible. These examples demonstrate the importance of thorough investigations of Legionnaires disease, even a single reported case, and the importance of notifying guests so that additional legionellosis cases can be identified during hotel outbreaks.

The investigation of these outbreaks also highlighted surveillance gaps, including delays in case identification and underreporting. Although Legionnaires disease is a nationally notifiable disease, hotel B’s outbreak was identified solely through a report from a member of the public. Legionnaires disease associated with a private vacation rental in USVI has been described previously (*7*); however, many travel-associated cases are likely missed among travelers who return home before becoming symptomatic. Including destinations in reports of travel-associated Legionnaires disease cases when notifying CDC is essential to improving multijurisdiction coordination that can help identify outbreaks and their sources (*8*).

This public health response underscores the importance of rapid reporting, environmental assessments, laboratory testing, and facility engagement in remediation to prevent additional illnesses. When investigating possible sources of *Legionella* outbreaks in tropical climates, public health officials should consider water systems without temperature regulation and alternative water storage systems, including cisterns.

## Footnotes

* Hotel A’s water supply system used both chlorinated municipal water and rainwater cisterns; water was filtered using sedimentation and filtration. Water from the cistern, although seldom used, was treated with chlorine and dispersed to hot water heaters before reaching guest rooms. On occasion, the cistern was filled by a water truck delivery.

† Positive results were considered presumptive until isolates were plated on buffered charcoal yeast extract, and their serogroups were identified to differentiate *L. pneumophila* serogroup 1 from other serogroups.

§ Legiolert reports in MPNs. The MPN of *L. pneumophila* colonies is based on reaction of *L pneumophila* with the enzyme substrate in the Legiolert test; after the number of positive wells is counted, an MPN table is used to determine the concentration in the original sample. The MPN method is considered scientifically equivalent to, or better than, the colony-forming unit plate method for determining concentration.

¶ Hotel B’s water supply system used chlorinated municipal water and stored the water in a cistern. No additional information was available.

** 45 C.F.R. part 46, 21 C.F.R. part 56; 42 U.S.C. Sect. 241(d); 5 U.S.C. Sect. 552a; 44 U.S.C. Sect. 3501 et seq.
